# Newborn Hearing Screening—Polish Experience: A Narrative Review

**DOI:** 10.3390/jcm14082789

**Published:** 2025-04-17

**Authors:** Krzysztof Szyfter, Wojciech Gawęcki, Witold Szyfter

**Affiliations:** 1 Institute of Human Genetics, Polish Academy of Sciences, 60-479 Poznan, Poland; 2 Department of Otolaryngology and Laryngological Oncology, Poznan University of Medical Sciences, 60-355 Poznan, Poland; wojgaw@interia.pl; 3 Faculty of Medicine, Prince Mieszko I Poznan Medical University of Applied Sciences, 60-320 Poznan, Poland; szyfterwitold@gmail.com

**Keywords:** hearing screening, newborn, hearing loss, UNHS

## Abstract

The Universal Neonatal Hearing Screening (UNHS) program is crucial for the early detection and treatment of hearing impairment in newborns. Poland has successfully implemented a nationwide UNHS program, adhering to international standards. Research indicates that hearing loss affects approximately 2–4 per 1000 infants, with sensorineural hearing loss being the most prevalent. Major risk factors include genetic alterations, craniofacial anomalies, prematurity, hyperbilirubinemia, and congenital infections such as cytomegalovirus. Despite the program’s success, challenges related to limited parental awareness and disparities in access highlight the need for continuous improvement in screening and follow-up procedures. Additionally, gene therapy is emerging as a promising treatment for hearing loss. While still experimental, gene therapy could become a key complementary treatment option in the future, offering new hope for those with hearing impairments.

## 1. Introduction

The sense of hearing is important during the early days of life for the development of speech, language and cognition. Hearing is equally vital for an individual’s development as well as for interaction with other people. Hearing loss is the most common congenital birth defect, affecting 1–3 infants per 1000 live births. On the other hand, understanding that early detection of medical defects enables faster treatment was central to the initiative focused on infant care. The Universal Newborn Hearing Screening Program (UNHS) was first established in developed countries. It is now mandatory in some countries and has been successfully implemented in others. Motivated by a common goal to diagnose and treat hearing impairment, Polish laryngologists and specialists in audiology and phoniatrics have sought to create a similar program in Poland, following the recommendations of The Joint Committee on Infant Hearing. The guidelines for achieving comparable results have been adopted [1]. The only remaining challenge has been securing the necessary funding.

## 2. Organization and Financial Conditions

To develop Universal Neonatal Hearing Screening, the organization required the involvement of all Polish neonatal units, including medical doctors, nurses, technicians and statisticians, as the main groups of employees. Just after the year 2000, Poland faced a difficult economic situation that forced limitations on governmental expenses in many fields including healthcare. The financial aspects of UNHS were presented by Fang et al. (2016) [2]. In their comprehensive study, the authors examined a correlation between Gross Domestic Product (GDP) and the efficiency of UNHS. Additionally, the study mentions that national programs are financed by government funds, insurance companies and charitable sources [2]. To our knowledge, Poland is the only country with a full program subsidized exclusively through charitable sponsorship. It has operated continuously at a national level for 22 years. The sponsor is the Great Orchestra of Christmas Charity Foundation. Each year, the Foundation focuses on a different healthcare initiative, supporting various programs (e.g., early oncological diagnosis for children, treatment and prevention of retinopathy in preterm infants, respiratory care for pregnant women, and providing medical equipment, including ambulances). UNHS is the longest-running and most stable program supported by the Orchestra. It should be noted that Mr. Jurek Owsiak has consistently been recognized in public opinion polls as one of the most respected and trusted individuals in Poland for many years.

## 3. General Results—Extent of Hearing Impairment Testing in Neonates

The Polish Universal Neonatal Hearing Screening Program (PUNHSP) has been operating for 22 years and focuses on early detection, diagnosis, and intervention for hearing disorders in children across three reference levels, each with specific actions. In Stage I, the technique known as Transient Evoked Otoacoustic Emission (TEOAE) is applied to all newborn children at 2–3 days of life (our routine targets the second day of a newborn’s life). Testing on the first day of life tends to produce false positive results as shown by Dimitriou et al. [3], who also suggest performing the test on the fourth day to minimize false results. Children who do not pass the TEOAE test are referred to Stage II for additional otoacoustic emission (OAE) and auditory brainstem response testing (ABR), in accordance to international standards [1,4,5,6]. The aim is to diagnose hearing impairment by the age of 3 months. If hearing impairment is confirmed, children are referred to Stage III. Prior to initiating treatment for referred children, the evaluation is extended to include potential factors contributing to hearing impairment. Specialized third referential level centers provide intervention, which includes fitting hearing aids, rehabilitation for speech and hearing, and regular monitoring. Intervention should begin by 6 months of age to support the child’s speech and language development. The program is managed by the Medical Coordinator’s Office, which oversees all levels, monitors results, and reports to the Foundation and relevant government bodies.

Our first reports covering the period from 2003 to 2006 were based on the examination of 1,392,427 children [6,7]. The coverage included 96.3% of all newborns delivered in Poland. Such coverage is comparable to the results of UNHS in other countries characterized differentially by financial and medical service status. The North-Reine province of Germany reported a coverage of 98.7% during the 2007–2016 period [8], Brazil 96.5% in 2013–2019 [9], Turkey 96.3% in 2004–2008 [10] and Saudi Arabia 92.6% in 2016–2021 [11]. Occasionally, authors highlight the large size of a country and the existence of hardly accessible regions that could contribute both to challenges and success [9,12].

Annually, 7.8 to 9.9% were sent for further testing. In our study [7], 2485 out of 1,392,427 tested babies were designated to Stage III. Finally, 312 children with profound, and 145 with severe sensorineural hearing loss were identified. Further studies within UNHS from 2002 to 2011, involving over 3.5 million babies, provided more information about the prevalence of hearing impairment in Polish children [13]. The total number of affected children was 5443, that is 2.97 per 1000 children, further divided into sensorineural (1.82 per 1000), conductive (0.79 per 1000) and mixed (0.36 per 1000) cases. The severity of hearing loss was dominantly in the range of 20–70 dB, with rare cases exceeding 70 dB and occasionally surpassing 90 dB. It is worth noting that there are differences concerning threshold value of hearing loss, which is typically set at 40 dB but is sometimes defined as 30 dB. Generally, hearing loss is categorized as follows: mild (21–40 dB HL), moderate (41–70 dB HL), severe (71–95 dB HL), and profound (>95 dB HL) [4,14,15].

In our subsequent study involving 109 out of 27,935 neonates, bilateral hearing impairment was found in 60.6% of cases, compared to 39.4% with a unilateral defect [16]. Further studies confirmed the above-cited results. A vast majority of studies estimate newborn hearing impairment to occur in 2–4 cases per 1000 infants [4,8,14,17]. The differing results do not appear to be related to geographic or ethnic differences but rather to the relatively small number of infants referred to Stage II. The latest report from the Polish Universal Neonatal Hearing Screening Program provides the following data: a total of 7 million children were registered in the program database by the end of 2021. In 2021, a total of 331,511 children were born in Poland, and 318,027 of them were registered in the PUNHSP, covering 96% of all newborns born that year. Of these, 312,833 (98.4%) were screened. The loss to follow-up rate was 1.6%, as 5194 children did not undergo the screening exam. Additionally, 8.4% (26,855 children) required Stage II follow-up, and of those referred, only 43.0% (11,622 children) attended secondary centers for further evaluation. Furthermore, 2.3% (343 children) diagnosed with hearing loss were referred to Stage III. The most common type of hearing loss in children was bilateral sensorineural hearing loss, which was diagnosed in 160 children in 2021 [18]. The data regarding the PUNHSP results from above-cited studies are summarized in Table 1.

## 4. Contributing Factors

The etiology of hearing impairment in newborns is not fully recognized and reports provide various categorizations of contributing factors. According to the study by Lammens et al. (2013) [19], the causal factors include: metabolic disorders (0.5%), infectious diseases (35.8%), congenital malformations (6.1%), and genetic alterations (19.8%). The cited results concerning neonatal hearing loss were derived from a study conducted on 569 neonates admitted to Stage III of UNHS [19]. Alternative results were drawn from the large 9-year study conducted in Beijing, China. According to the authors, the main risk factors included craniofacial anomalies, length of stay in the neonatal intensive care unit and birth weight less than 1500 g [15]. There are few studies attempting to link the type of hearing deficit with the type of hearing impairment. However, the report from the Belgium Program analyzing 545 infants referred to Stage II established genetic etiology (32.1%) as the most common factor in bilateral sensorineural hearing loss. For unilateral sensorineural hearing loss, anatomical abnormality (26.5%) was the leading factor [20].

A few other factors should be considered next. It seems obvious that craniofacial anomalies are connected with conductive hearing loss, as shown in a large Chinese study involving 1839 infants referred to Stage II [15]. Genetic determination of impairment was the most frequent in bilaterals, while anatomical abnormalities were found in babies with unilateral hearing loss [19].

A positive family history of hearing impairment is also a strong risk factor in each type group [4,5]. The study on children referred after UNHS in France (n = 1705) has shown family history of deafness as a leading cause of congenital hearing impairment. Family history of deafness was followed by syndromic deafness, which in turn indicates genetic factors [21]. The role of genetics will be discussed separately.

Further analysis within UNHS focused on the reinvestigation of 27,935 children referred to Stage III. Of these, 109 children were diagnosed with hearing impairment recognized as sensorineural (n = 38), conductive (n = 56), and mixed (n = 15) type. Bilateral hearing deficit was the most common condition [16]. A careful analysis of demographic variables, family history of hearing deficit, and the analysis of medical records contributed to the association of certain conditions with hearing deficit. The most common association in our study group linked hearing loss with hyperbilirubinemia requiring phototherapy. This finding does not have a counterpart in other studies and remains difficult to explain. It was observed in all types of hearing deficit and exceeded 50%. Hyperbilirubinemia might be the cause of auditory neuropathy spectrum disorders [4,5,22]. An impact of hyperbilirubinemia on neurodevelopment was established. The preventive assessment of neonatal hyperbilirubinemia was suggested [23].

Prematurely born children (n = 11,438) were studied separately. It was expected that children born before the regular gestation term did not complete their development and, as a result, could accumulate risk factors of hearing impairment. Hearing deficit increased progressively as gestational age decreased. The group was then divided into subgroups born before 28 weeks of gestational age (n = 2884) and within 29–32 weeks (n = 8554). Hearing impairment prevalence was considerably higher in the first group, regardless of the type of hearing deficit. Upon examining selected risk factors, it was found that very low birth weight, mechanical ventilation for at least 5 days, and ototoxic medication were significantly more frequent in the group born at <28 weeks. Craniofacial anomalies were also more frequent but did not reach statistical significance. Hence, a general conclusion is that hearing impairment is a severe consequence of prematurity [17].

Both hyperbilirubinemia and craniofacial anomaly have been presented and discussed among primary statistically significant risk factors for hearing impairment in a systematic review by Alhazmi [22].

Another contributing factor taken into account is congenital infection with cytomegalovirus (CMV), either alone or as part of TORCH syndrome. The latter is caused by an in-utero infection. There are various possible effects of CMV infection, including sensorineural hearing impairment. CMV infection may cause immediate or late-onset sensorineural hearing loss, which can be missed in standard UNHS. Recently, CMV has become the most common congenital infection in developed countries and is considered an important non-genetic contributing factor to hearing loss (HL) [24].

In our study, which aimed to identify risk factors in a group of 109 referred newborns, CMV infection was detected in four cases with sensorineural HL and one case with conductive hearing deficit. This does not exclude the possibility of further late-onset HL manifestation [16].

Perinatal ototoxic treatment is another hearing impairment contributing factor, falling between the side effects of medical treatment and genetics. While it is considered a contributing factor, its prevalence does not rank among the top causes [4,16]. It is likely that during interview, it could be covered by a family history of hearing deficit. In our study of 5601 infants referred by UNHS, hearing impairment was found in 436 cases (7.78%) attributed to ototoxic medications [6]. This result was later confirmed in a larger-scale study (n = 27,935) [16]. Ototoxic treatment is commonly associated with the application of aminoglycoside drugs with antibacterial activity, with gentamycin being the most frequently prescribed. Our contribution to the field was a study on COI/tRNA(Ser(UCN)) genes in 250 unrelated Polish subjects with hearing impairment. The incidence of m.7444 G>A substitution was estimated at 1.6% (4/250), however variable penetrance of hearing loss, age of onset, and hearing thresholds among m.7444 G>A carriers was observed. Two subjects had a positive history of aminoglycoside exposure, and one of them carried both the m.7444 G>A and 12S rRNA m.1555 A>G mutations. The findings strongly suggest a possible role of the m.7445 A>G mutation in susceptibility to aminoglycoside-induced hearing loss [25]. A very low prevalence of tRNA(ser(UCN)) mutations was shown in a study involving 479 samples of maternal DNA and paired neonatal specimens. Maternal and neonatal subjects had high aminoglycoside exposure. All samples tested were negative for the m.1555 A>G and m.1494 C>T mutations [26]. Our conclusion was that m.7444 G>A mutation alone is not sufficient to produce a clinical phenotype and additional modifier factors are required for the pathogenic manifestation of the m.7444 G>A substitution. In this way, we received indirect support for our supposition through negation [25]. A systematic review based on 31 publications still confirmed that aminoglycosides are associated with the risk of irreversible sensorineural hearing loss. The mutations m.1555 A>G and m.1494 C>T have the strongest impact on the development of aminoglycoside-related SNHL [27]. One must remember that ototoxic and nephrotoxic drugs are frequently used in neonatal intensive care. A prospective cross-sectional study conducted at Italian and Spanish neonatal units have shown the use of aminoglycosides, followed by vancomycin, loop diuretics and ibuprofen. The authors emphasized the need for standardization of procedures in accordance with pharmacogenetics [28].

## 5. Significance of Genetic Background

The estimated impact of genetic factors on hearing impairment in newborns varies significantly. A well-written review by Morton and Nance [4] attributes 44% of hearing loss at birth to syndromic or non-syndromic deafness, 21% to *GJB2* gene mutation, and 3% to Pendred syndrome. According to the authors, the vast majority of HL is determined by genetic factors. Another review (2013) presented a similar estimate, including 47% of cases connected with a family history of deafness and 41% with syndromic deafness [29]. Once again, a dominant role of genetic factors is highlighted. However, Lammens at al. (2013), in their review, estimated the contribution of genetics to the HL etiology at only 20% [19]. A more recent review presenting the results of newborn hearing screening in China estimated the influence of genetic factors to be over 50% [30].

Differences, to some extent, can be explained by the method of estimation. In principle, some studies focus solely on classical Mendelian genetics, while others perform experiments in the field of molecular genetics. Examples taken from the original papers primarily concern the use of Mendelian genetics. The etiology of newborn HL was tested in 306,285 Australian children, with genetic causes found in 26,8% of cases [31]. Another study involving 429 American pediatric HL cases (not necessarily newborns) of mixed origin were tested for genetic causes. The testing included family history, karyotyping, analysis of selected ethnicity-related genes (such as *GJB2*, *GJB6*), a Pendred syndrome panel (*SLC26A4* gene), and others. Genetic causes of HL were identified in 34% of cases, later categorized into non-syndromic and syndromic HL (46,59% and 32,41%, respectively). In the non-syndromic group, 65% of cases were carriers of the mutated *GJB2* gene. The most frequent gene in the syndromic group (22%) was the *SLC26A4* gene [32]. The significance of the *GJB2* gene was further studied in 924 individuals referred through the UNHS program, with the study extended to include family members. A total of 18 pathological mutations of *GJB2* were identified. Hearing status was found to depend on the type of mutation (position, heterozygous, homozygous). This indicates that HL associated with *GJB2* mutations may not always be congenital at birth [29]. Variants of the most frequent genes associated with HL at birth were pooled and analyzed in China. The initial sample size derived from the UNHS program was 2,161,984 newborns. The most prevalent deafness gene among Chinese neonates was the *GJB2* c.235delC variant. Another *GJB2* gene variant that was tested is c.299_300delAT. The second most frequent gene was *SLC26A4* (variant c. 919-2 A>G). Significant regional distribution characteristics were observed [33]. These findings were fully supported by two large Chinese studies. The study, which included cases referred to Stage II (n = 76,460 newborns from Beijing), investigated 15 variants in four genes: *GJB2*, *SLC26A4*, mtDNA *12S rRNA*, and *GJB3*. The most frequent mutant allele was c.235delC in the *GJB2* gene, followed by c.919-2 A>G in the *SLC26A4* gene [34]. Another Chinese study presented genetic screening of 15 hearing loss genes in 2369 neonates referred for Stage II, selected from 77,647 newborns initially tested at Stage I. The carrier frequency of *GJB2* gene mutations was the highest, followed by *SLC26A4* mutations. The latter were found to be associated with late-onset hearing loss. The authors emphasize the importance of genetic screening as a valuable addition to conventional hearing screening [35]. The dominant role of mutations in the *GJB2* and *SLC26A4* genes has been found and confirmed in numerous publications.

The significance of genetic factors as a risk agent for HL was not the focus of our studies. However, in terms of Mendelian genetics, a family history of HL was found in 5.3% of cases, and congenital anomalies were much more frequent [16]. A genetic background was also identified in a group of 27,935 infants referred by the program. This included 15 cases of Down syndrome and two cases with a positive family history of hearing deficit [16].

## 6. Questions, Problems, and Difficulties

The aim was to implement UNHS in all countries to access the global character of neonatal hearing screening. The expected benefits are clear, but there have been obstacles such as insufficient funding, crisis and war. According to the WHO, UNHS covered 158 countries in 2022, leaving 38% of the word’s newborns without hearing screening [36]. Even when the program is implemented in a given country or territory, it does not guarantee full coverage due to geographic, economic and social reasons [6,7,8,9,10,11]. Disparities have been studied in detail based on the American experience [37]. While working within the program, we observed a significant absence of children referred to Stage II. This absence, reaching 51%, was associated with parents’ low education levels and their lack of awareness that the risk of developing HL increases sixfold compared to the risk at birth [37]. Another study within the Spanish UNHS, which focused on HL beyond the neonatal age, also indicated an increase in the number of children with hearing impairment, from 1.5 per 1000 to 2.7 per 1000 in the postnatal stage [38]. Data from a meta-analysis of 19 studies, including 169,238 infants, highlighted the impact of infant and maternal sociodemographic factors. For infants, factors included low birth weight, racial minority status, rural residence, and others. Racial minority status, however, is not a relevant factor in Poland, where the population is predominantly Caucasian. Maternal factors included low education, young maternal age, unmarried status, and tobacco smoking [39]. The results of this analysis align with our observations [40]. Further efforts from healthcare providers are needed to ensure the success of the program and the well-being of infants.

Another issue is strictly medical and was already discussed when presenting cCMV infection [16,24]. It can cause late-onset SNHL, and the hearing impairment may remain undetectable, particularly in cases of progressive SNHL. Hearing loss tends to occur earlier in symptomatic cases than in asymptomatic ones [41].

The final obstacle to be discussed is the COVID-19 pandemic in 2020–2021. While some units were suspended, the program itself was not discontinued. Telephone communication and telemedicine were intended to support its regular activities [42]. A report from the Brazilian part of the UNHS mentioned a slight decrease in several contributing factors, leading to a small drop in referrals for the second stage [43]. American studies on 30,773 newborns have shown no differences in hearing impairment detection in infants before and during the COVID-19 pandemic [44].

## 7. Perspectives

Determining hearing impairment and, hopefully, its causal factors necessitates the initiation of treatment (Stage III). In general, a variety of approaches are available, including pharmacological or surgical treatment, hearing aids, cochlear implants, bone conduction implants, and gene therapy.

In cases of confirmed bilateral profound hearing loss with no benefit from hearing aids, cochlear implants are the commonly accepted and well-established solution. Currently, it is recommended that implantation be performed immediately after the completion of the first year of life [45], although in some centers, this procedure is performed even earlier [46]. The importance of early implantation for speech and language development has been well-documented for many years [47,48]. The 10-year summary of PUNHSP, which includes data from 23 centers, shows that 58.2% of children diagnosed with hearing loss are referred for hearing aid fitting, 34% are referred for surgical treatment, and 7.8% require rehabilitation [49]. Unfortunately, significant disadvantages of both hearing aids and hearing implants include relatively high level of background noise and the need for regular adjustments as the child grows.

A real hope for children with HL lies in a molecular treatment known as gene therapy. Restoration of hearing has been demonstrated in a mouse model using more than 20 genes [50]. In humans, technical challenges remain, such as identifying the gene responsible for the defect, delivering the correct gene to its proper location, and replacing the “wrong” gene with the correct one. These processes are under advanced study. The transportation of the proper gene to the target cell (inner hair cell) is managed by modified viruses [51]. The most recognized gene in this context is the *OTOF* gene, also known as *DFNB9*, a coding calcium-binding protein otoferlin expressed in inner hair cells. Replacing the *OTOF* gene in inner ear hair cells has the potential to restore hearing. A Chinese laboratory reported successful hearing restoration in five out of six children [52]. Other laboratories in the USA and Israel are working along similar lines. Therefore, gene therapy is rapidly advancing and may soon reach its goal.

## Figures and Tables

**Table 1:** Summary of the results of the PUNHSP referenced in the manuscript. The percentages of children referred to Stage II and those diagnosed with HL are given with respect to the number of children screened in Stage I.

PUNHSP	Time Period and the Relevant Study			
	2003–2006 [7]	2002–2011 [13]	2010–2014 [16]	2021 [18]
Number of children screened (Stage I)	1,392,427 (96.3%)	3,322,349 (96%)	27,935 (98%)	312,833 (98.4%)
Number of children referred to Stage II	178,821 (12.8%)	292,375 (8.8%)	N/A	26,855 (8.6%)
Number of diagnosed HL cases	2485 (0.18%)	5443 (0.16%)	109 (0.39%)	343 (0.11%)

## Abbreviations

The following abbreviations are used in this manuscript:

UNHS	Universal Neonatal Hearing Screening
TEOAE	transient evoked otoacoustic emission
OAE	otoacoustic emission
ABR	auditory brainstem response
PUNHSP	Polish Universal Hearing Screening Program
CMV	cytomegalovirus
SNHL	sensorineural hearing loss
HL	hearing loss
